# CircRNAs in Immuno-Metabolic Reprogramming of Chordoma Cancer: Molecular Crosstalk and Therapeutic Potential

**DOI:** 10.3390/ijms27020990

**Published:** 2026-01-19

**Authors:** Negar Taghavi Pourianazar

**Affiliations:** Medical Laboratory Techniques, Istanbul Aydin University, 34295 Istanbul, Turkey; negartaghavi22@gmail.com or negarpourianazar@aydin.edu.tr

**Keywords:** immunometabolism, chordoma, circRNAs, metabolic reprogramming, immune evasion

## Abstract

Slow-growing and locally invasive, chordoma is a rare malignant bone tumor, with a reported annual worldwide incidence of 0.08 per 100,000 cases. It accounts for about 3 percent of all bone tumors and about 20 percent of primary spinal tumors. The incidence rates vary between countries and races, with white/Caucasian males in the 5th or 6th decade of life having a higher prevalence. Chordoma poses significant challenges because of its high recurrence rate and resistance to several standard treatment techniques. All cancers, including chordomas, have altered energy metabolism processes that contribute to their unchecked growth and survival. The significance of non-coding RNAs, particularly circular RNAs (circRNAs), as key regulators at the intersection of cellular metabolism and immune function has been highlighted by recent discoveries. By focusing on important glycolytic enzymes in tumor cells and altering metabolic reprogramming pathways, CircRNAs can influence cancer metabolic adaptability. Furthermore, via influencing immune cell functions as immunological checkpoint signaling and macrophage polarization, circRNAs influence immune evasion in the tumor microenvironment. These frequently happen via regulating important pathway signals, like PI3K/AKT/mTOR and NRF2, or by processes like miRNA sponging, creating a tumor microenvironment that is immunosuppressive and metabolically friendly. The translational pathway of circRNA-targeted therapeutics is promoted as a developing pharmacological entity in this review, which also highlights recent information on the control of circRNA-mediated immunometabolism in chordoma and examines numerous important molecular axes. There are promising opportunities to develop novel precision treatments for chordoma by considering circRNAs as dual regulators of immunological and metabolic networks.

## 1. Introduction

### 1.1. Chordoma: Characteristics and Pathophysiology

Chordoma is an uncommon malignant bone tumor that grows slowly and spreads locally. It starts from the remains of the basic notochord [1]. The sacrum/coccyx and the base of the skull are the most often affected areas, and it mostly affects the axial skeleton. Despite being regarded as low-grade tumors, chordomas have a dismal prognosis because of their high recurrence rates, potential for metastasis, and local aggressiveness [2]. The molecular foundations of chordoma have been further clarified by recent research, which has also highlighted the disease’s distinct cellular features and treatment difficulties. Since these embryonic remains are normally regulated for apoptosis, the notochordal origin of chordoma is crucial because they have the potential to change malignantly and cause tumor initiation [3]. This metamorphosis is frequently associated with particular genetic and epigenetic changes that promote unchecked growth and survival. Due to chordoma’s slow growth rate, diagnosis is frequently delayed, allowing the tumor to develop to a large and invasive size before being discovered. Since total surgical excision is frequently difficult, particularly in crucial anatomical regions like the base of the skull, this local invasiveness plays a significant role in its high recurrence rates [2].

### 1.2. Pathophysiology

**Figure 1:**
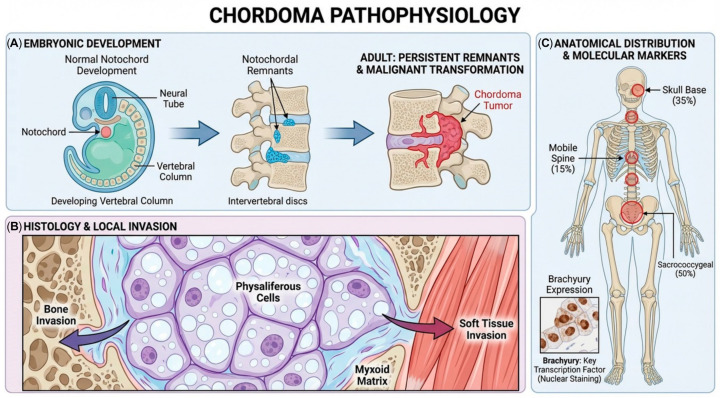
An illustration of chordoma’s pathophysiology. This figure illustrates the key aspects of chordoma development and its clinical features. (**A**) The process begins with persistent notochordal remnants from embryonic development that undergo malignant transformation into chordoma. (**B**) Histologically, the tumor is characterized by physaliferous cells with vacuolated cytoplasm and shows aggressive local invasion into bone and soft tissue. (**C**) Chordomas are primarily located in the skull base (35%), mobile spine (15%), and sacrococcygeal region (50%) and are defined by the overexpression of the transcription factor brachyury, a key diagnostic marker.

Notochordal cells that continue to grow abnormally after embryonic development are recognized to be the source of chordomas; however, the exact mechanisms causing them are still being investigated [3]. Normally, these cells retreat, but in chordoma, they change malignantly. Due to the tumor’s slow growth, detection is sometimes delayed, which permits it to spread far and infiltrate nearby tissues, such as soft tissue and bone (Figure 1). Depending on the location of the tumor, this local invasiveness can cause serious morbidity by compressing important structures, resulting in symptoms including pain, neurological impairments (e.g., weakness, numbness, bladder/bowel malfunction), and headaches. It is also becoming more well acknowledged that the chordoma tumor microenvironment (TME) plays a crucial role in determining the course of the tumor and the effectiveness of treatment. Immune cells, stromal cells, and extracellular matrix constituents interact intricately to create an immunosuppressive environment, which is its defining feature [4,5].

Chordomas are distinguished genetically by certain molecular changes. Consistently overexpressed in chordoma cells, brachyury is a transcription factor essential for notochord formation and is regarded as a possible therapeutic target as well as a critical diagnostic marker [6,7]. Recent studies have also discovered recurring genetic abnormalities that contribute to chordoma pathogenesis and treatment resistance, such as mutations in genes related to signaling pathways (e.g., PI3K/AKT/mTOR pathway components) and cell cycle regulation (e.g., CDKN2A) [8,9,10]. Histone changes and DNA methylation are examples of epigenetic modifications that are important in controlling gene expression and promoting the growth of chordomas [11].

### 1.3. Circular RNAs in Cancer Immunometabolism

Circular RNAs (circRNAs), a kind of endogenous non-coding RNA, are more stable than linear RNAs due to their covalently closed loop structure [12,13,14]. New evidence demonstrates their critical significance in various biological processes, such as carcinogenesis, tumor development, and TME modulation. Remarkably, circRNAs have a significant role in immunometabolism, the intersection of cellular metabolism and immune activity [15]. These general circRNA mechanisms have been characterized in various cancer types; however, whether these mechanisms operate similarly in chordoma remains to be determined.

### 1.4. Role in Metabolic Reprogramming

**Figure 2:**
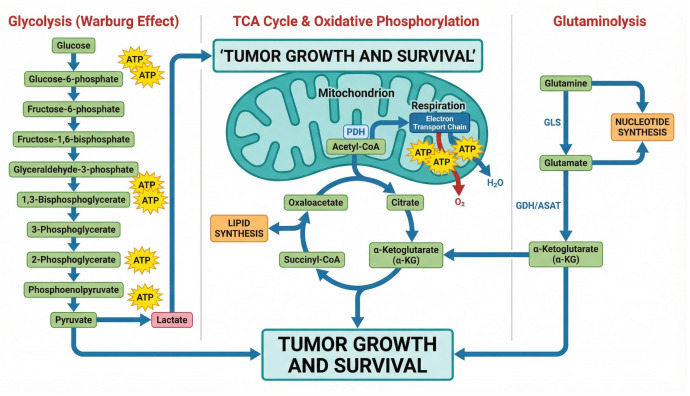
Schematic illustrating glycolysis, TCA cycle, and glutaminolysis pathways that support tumor growth and survival through ATP production, biosynthetic precursor generation, and lactate-mediated immune evasion; key regulatory enzymes (PDH, GLS) are potential targets for circRNA-based therapeutic intervention. PDH, Pyruvate dehydrogenase; GLS, Glutaminas; TCA cycle, Tricarboxylic acid cycle.

Cancer cells exhibit altered metabolic pathways to support their rapid proliferation and survival, a phenomenon known as metabolic reprogramming. This metabolic plasticity allows cancer cells to adapt to fluctuating nutrient availability and hypoxic conditions within the tumor microenvironment. CircRNAs have been identified as key regulators of these metabolic adaptations, influencing various aspects of glucose, lipid, and kinase (PFK), by a variety of circRand amino acid metabolism within tumor cells [16]. For example, the Warburg effect, a metabolic shift in which cancer cells preferentially rely on glycolysis even in the presence of oxygen, is facilitated by the regulation of glycolytic enzymes, such as hexokinase 2 (HK2) and phosphofructokinase (PFK), by a variety of circRNAs [17,18]. This metabolic reprogramming gives cancer cells the building materials they need to divide and expand quickly, as well as the intermediates they need for anabolic functions. In addition to glycolysis, circRNAs also affect other metabolic processes that are essential for maintaining the high energy requirements of cancer cells in growth, such as glutaminolysis, fatty acid synthesis, and oxidative phosphorylation (Figure 2) [19]. In chordoma specifically, whether circRNAs regulate glycolytic enzymes or other metabolic pathways remains unknown. While chordoma cells likely undergo metabolic reprogramming to support their growth, the specific circRNAs involved and the particular metabolic pathways affected have not been systematically characterized. By analogy with other cancers, circRNAs may regulate chordoma cell metabolism, but direct evidence from chordoma-derived cells or tissues is currently lacking.

### 1.5. Role in Immune Regulation

**Figure 3:**
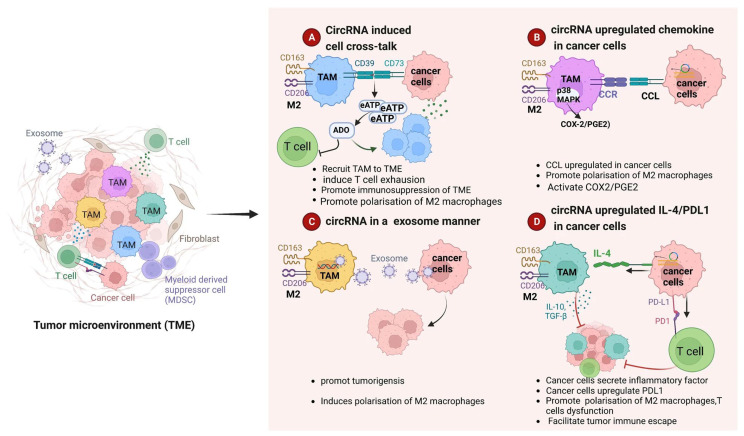
Schematic illustration of regulating activities of circRNAs in the tumor microenvironment. (**A**) CircRNAs create an immunosuppressive tumor microenvironment (TME) by mediating interaction between tumor cells and macrophages, inducing M2 macrophage polarization, and impairing T cell activity. (**B**) CircRNAs cause M2 macrophage polarization and increase chemokine expression in tumor cells. (**C**) To improve M2 macrophage polarization, tumor cells release circRNA-containing exosomes into macrophages. (**D**) CircRNAs encourage tumor cells to secrete immunosuppressive and inflammatory substances in order to attract and polarize M2 macrophages and inhibit T lymphocytes [35].

CircRNAs have a significant influence on immunological responses within the TME, which aids in immune evasion, in addition to their direct effects on tumor cell metabolism [20]. They have the ability to affect the polarization and activity of different immune cells, such as T cells, natural killer (NK) cells, and macrophages. According to recent data, circRNAs usually do not interact with immune cells by directly attaching to ligands at the membrane surface. Instead, extracellular vesicles—specifically, exosomes released by tumor cells and adjacent stromal elements—are the primary means by which circRNAs are transported to recipient cells. Through membrane fusion or endocytic uptake, macrophages swallow these vesicles, allowing circRNAs to build up inside cells. For instance, it has been demonstrated that specific circRNAs alter macrophage polarization toward an M2-like phenotype, which is linked to angiogenesis, immunosuppression, and tumor development [21,22]. By releasing anti-inflammatory cytokines and encouraging tumor growth, these M2 macrophages help to create an immunosuppressive microenvironment. Additionally, circRNAs can control immune checkpoint signaling pathways, including PD-1/PD-L1 and CTLA-4, which are important targets in cancer immunotherapy, and cause T cell depletion and malfunction, which reduces their anti-tumor efficacy (Figure 3) [23,24]. In chordoma specifically, the TME is characterized by M2 macrophage dominance and T cell exhaustion with elevated expression of checkpoint molecules such as PD-1 and CTLA-4 [25]. By analogy with other cancers, circRNAs may drive this immunosuppressive phenotype in chordoma. However, direct evidence that circRNAs regulate macrophage polarization or T cell exhaustion in chordoma-derived cells or tissues is currently absent. The specific circRNAs involved in chordoma immune suppression have not been identified, and the mechanistic links between circRNA expression and immune cell phenotypes in chordoma remain unknown.

In other malignancies, circRNAs regulate immune cell phenotype through multiple mechanisms. Regarding T cell exhaustion, circPVT1 has been shown to promote T cell exhaustion in melanoma by upregulating PD-1 expression through a circPVT1/miR-125b/PD-1 regulatory axis [26,27]. In non-small cell lung cancer, circDENND2D regulates T cell exhaustion through direct interaction with the PD-1 signaling pathway, contributing to the immunosuppressive tumor microenvironment [28].

The regulation of M2 macrophage polarization by circRNAs has also been extensively documented. CircATP8A1 promotes M2 macrophage polarization in gastric cancer by acting as a miRNA sponge for miR-1-3p, thereby preventing miR-1-3p-mediated suppression of STAT6, which is a master regulator of M2 macrophage differentiation [29]. In gastric cancer, CircRNA_102191 enhances M2 macrophage polarization by stabilizing IL-4 receptor mRNA, which promotes IL-4-mediated M2 differentiation and the associated immunosuppressive phenotype [30]. Additionally, in colorectal cancer, circPCLE1 regulates tumor-associated macrophage M2 polarization through competitive adsorption of miR-485-5p to mediate ACTG1 expression, influencing the balance between anti-tumor immunity and immunosuppressive microenvironment [31]. Furthermore, in colorectal cancer, circMERTK regulates tumor-associated macrophage immunosuppressive activity through the miR-125a-3p/IL-10 axis, influencing CD8+ T cell apoptosis and facilitating immune evasion [32].

Beyond T cell and macrophage regulation, circRNAs also directly modulate immune checkpoint pathways. Circ-HSP90A directly enhances PD-L1 expression in lung cancer by promoting PD-L1 mRNA stability, thereby increasing the immunosuppressive signal in the tumor microenvironment [33]. Similarly, circCTLA-4 regulates CTLA-4 expression in melanoma through a circRNA-mediated mechanism, contributing to T cell exhaustion and immune evasion [34].

These findings collectively establish that circRNAs can regulate immune cell phenotype and checkpoint expression through diverse molecular mechanisms, providing a strong mechanistic rationale for investigating similar roles in chordoma. However, such regulation has not been experimentally tested in chordoma-specific contexts, representing a critical knowledge gap that must be addressed through targeted experimental investigation.

By acting as miRNA sponges, interacting with RNA-binding proteins, or directly influencing gene expression, circRNAs can orchestrate a complex network that leads to immune tolerance and evasion, thereby allowing cancer cells to escape immune surveillance and proliferate unchecked [36].

### 1.6. Molecular Crosstalk in Immunometabolism

The TME is shaped by the intricate and varied interactions among circRNAs, metabolism, and immunology that create a complex molecular crosstalk. CircRNAs are essential for integrating immunological responses with metabolic cues. CircRNAs, for instance, have the ability to alter important signaling pathways that are essential to immune cell function and cellular metabolism, including PI3K/AKT/mTOR and NRF2 [37].

A master regulator of cell growth, proliferation, and survival, the PI3K/AKT/mTOR pathway is frequently aberrantly activated in cancer, which affects immune cell differentiation and function and frequently results in increased glycolysis and lipid production. Likewise, NRF2 is a transcription factor that affects immune cell responses as well as tumor cell survival and is essential for metabolic adaptability and antioxidant defense. By altering these pathways, circRNAs can suppress anti-tumor immune responses in a number of ways, such as encouraging immune cell fatigue or anergy, while also giving tumor cells a metabolically favorable environment that gives them plenty of energy and building blocks [38]. CircRNAs’ dual regulatory ability makes them important participants in the immunometabolic reprogramming of cancer, providing fresh perspectives on the pathophysiology of the disease and possible treatment approaches. This is especially true for chordoma, where complex interactions are likely to have a significant impact on the course of the disease and resistance to treatment.

In chordoma specifically, the PI3K/AKT/mTOR and NRF2 pathways are known to be aberrantly activated and may contribute to both metabolic reprogramming and immune suppression [39,40]. By analogy with other cancers, circRNAs may regulate these pathways in chordoma. However, whether circRNAs directly modulate PI3K/AKT/mTOR or NRF2 signaling in chordoma cells has not been demonstrated. The specific molecular crosstalk between circRNAs, metabolism, and immunity in the chordoma TME remains largely unexplored.

### 1.7. Specific CircRNA Mechanisms in Chordoma: Bridging to Immunometabolism

Research on circRNAs in chordoma’s immunometabolic reprogramming is still in its early stages. To date, there are no chordoma-specific datasets reporting circRNA expression in immune cell subsets. Therefore, proposed links between circRNAs and T-cell exhaustion or M2 macrophage polarization currently rely on mechanistic evidence from other malignancies (e.g., melanoma, breast cancer, gastric cancer, hepatocellular carcinoma) and remain hypothesis-driven rather than experimentally confirmed in chordoma. However, mechanistic insights from studies in other cancers provide a foundation for proposing hypothetical models of how circRNAs might regulate chordoma’s metabolic adaptations and immune evasion tactics. Given chordoma’s distinct microenvironment—characterized by slow growth, local invasiveness, and resistance to standard therapy [41]—circRNAs may play regulatory roles in these processes, but such roles remain to be experimentally validated in chordoma-specific contexts. This section first summarizes experimentally demonstrated circRNA functions in chordoma, then presents hypothetical immunometabolic models requiring validation, and finally outlines proposed experimental approaches to test these hypotheses.

### 1.8. Experimentally Validated CircRNA Mechanisms in Chordoma

CircTEAD1 represents the most robustly validated circRNA mechanism in chordoma to date. The discovery of circTEAD1 as a major oncogenic driver is significant because it establishes the first direct connection between a particular circRNA and the Hippo/Yap1 oncogenic pathway in chordoma. By stabilizing Yap1 mRNA, recent work has shown that circTEAD1 is elevated in chordomas and stimulates carcinogenesis. This is accomplished by a m6A-dependent mechanism in which circTEAD1’s cytoplasmic export and the creation of a circTEAD1/IGF2BP3/Yap1 mRNA complex are facilitated by METTL3-mediated m6A modification of circTEAD1. This discovery is significant because it establishes the first direct connection between a particular circRNA and the Hippo/Yap1 oncogenic pathway in chordoma, making it possible to study other circRNAs with comparable mechanisms and offering a tangible illustration of how circRNAs function in this disease. High-throughput circRNA sequencing of three chordoma–normal tissue pairs identified 1461 upregulated circRNAs, of which 17 were associated with the Hippo pathway. RT-qPCR validation identified circTEAD1 (hsa-circ_TEAD1_0016, 283 bp, formed by backsplicing between exons 7 and 10) as the most consistently upregulated candidate. Multiple complementary approaches confirmed circTEAD1’s circular structure and stability: Sanger sequencing validated the junction site, RNase R digestion demonstrated resistance to degradation, actinomycin D assays showed extended half-life compared to linear TEAD1, and FISH confirmed predominant cytoplasmic localization.

Functional studies in two chordoma cell lines (U-CH1 and U-CH2) using circTEAD1 knockdown and overexpression constructs demonstrated that circTEAD1 significantly promoted cell proliferation, invasion, and migration across multiple assays (colony formation, CCK-8, Transwell, and wound healing). Mechanistically, RNA pull-down followed by mass spectrometry identified METTL3 and IGF2BP3 as key circTEAD1-binding proteins, confirmed by RIP assays. Luciferase reporter assays, RT-qPCR, and Western blotting collectively revealed that Yap1 mRNA is the direct target of circTEAD1.

The regulatory mechanism involves m6A-dependent export of circTEAD1. SRAMP prediction and MeRIP assays identified two high-confidence m6A modification sites within circTEAD1. Mutation of these m6A motifs demonstrated that m6A modification is critical for circTEAD1 function. METTL3 knockdown elevated nuclear circTEAD1, while overexpression promoted cytoplasmic export, establishing that METTL3-mediated m6A modification facilitates circTEAD1’s cytoplasmic localization. Treatment with 3-Deazaadenosine (DAA), an m6A methylation inhibitor, further confirmed this mechanism.

CircTEAD1 exerts its oncogenic effects through formation of a ternary complex with IGF2BP3 and Yap1 mRNA. RIP assays and domain mapping identified the KH1-3 tri-domain of IGF2BP3 as the specific binding site for both circTEAD1 and Yap1 mRNA. This ternary complex stabilizes Yap1 mRNA and activates the Hippo signaling pathway, driving chordoma tumorigenesis. In vivo tumor formation assays validated this circTEAD1/Yap1 regulatory relationship, and elevated circTEAD1 expression emerged as a prognostic indicator correlating with worse disease-free survival [34].

However, important limitations must be acknowledged. The initial screening used only three tissue pairs, limiting generalizability of circRNA expression patterns. Functional studies were confined to two cell lines, which may not represent chordoma’s molecular heterogeneity and may have acquired culture-induced alterations. In vivo studies employed tumor formation assays but lacked orthotopic models recapitulating chordoma’s anatomical complexity (skull base or sacrococcygeal locations) or its native immunosuppressive microenvironment. Critically, the studies focused on cell-autonomous properties and did not investigate circTEAD1’s potential roles in immune regulation or metabolic reprogramming—mechanisms central to understanding chordoma’s “immune-cold” phenotype. Additionally, validation in independent chordoma cohorts, patient-derived xenografts, or organoid models remains absent. These limitations underscore the need for larger-scale studies in anatomically accurate models, investigation of circTEAD1’s immunometabolic functions, and independent cohort validation to establish circTEAD1 as a robust therapeutic target in chordoma.

To transition these hypothetical models into validated knowledge, the following experimental approaches are essential:

*Metabolic Flux Analysis:* Direct measurement of metabolic activity in chordoma cells with circRNA manipulation is required. Seahorse XF Analyzer assays should be performed to measure glycolytic rate and oxidative phosphorylation capacity in U-CH1 and U-CH2 cells following circTEAD1 knockdown or overexpression, and in cells with knockdown of candidate circRNAs proposed to regulate glycolytic or glutaminolytic enzymes. These assays would directly test whether circRNA manipulation alters metabolic phenotype. Additionally, 13C-labeled metabolic tracing using glucose or glutamine should be performed to quantify flux through glycolysis and the TCA cycle, providing mechanistic insight into circRNA-mediated metabolic reprogramming.
*Immune Co-culture Experiments:* To test whether circRNAs modulate the chordoma immune microenvironment, in vitro co-culture experiments should be established using chordoma cells (U-CH1, U-CH2) co-cultured with primary human macrophages or T cells. In these co-cultures, circRNA expression should be manipulated (via siRNA knockdown or overexpression vectors) and immune cell phenotype assessed through flow cytometry for markers of M1/M2 polarization (CD86, CD163, IL-10, TNF-α for macrophages; PD-1, TIM-3, IFN-γ, IL-2 for T cells) and functional assays measuring T cell proliferation and cytokine production. These experiments would directly test whether circRNAs regulate immune cell phenotype in the chordoma context.
*Single-Cell RNA-Sequencing of Immune Cells:* Single-cell RNA-seq (scRNA-seq) should be performed on immune cell populations isolated from chordoma xenografts with circRNA manipulation (circTEAD1 knockdown vs. control). This would reveal which circRNAs are expressed in specific immune cell subsets (macrophages, T cells, B cells, dendritic cells) and how circRNA manipulation alters immune cell transcriptomes and functional states. Integration of circRNA expression data with immune cell phenotype markers would identify candidate circRNA-immune cell interactions for further validation.
*In Vivo Xenograft Studies with Immune Profiling:* Comprehensive in vivo validation requires xenograft studies in immunocompetent mice (syngeneic models if available, or humanized immune reconstitution models) with circRNA manipulation combined with detailed immune profiling. Tumors should be analyzed at multiple timepoints for: (1) circRNA expression levels via RT-qPCR; (2) metabolic markers via immunohistochemistry (HK2, GLUT1, LDHA for glycolysis; GLS for glutaminolysis); (3) immune cell composition via flow cytometry (CD8+ T cells, Foxp3+ Tregs, CD11b+ macrophages, M1 vs. M2 markers); (4) immune checkpoint expression (PD-L1, CTLA-4); and (5) functional immune responses (T cell activation markers, cytokine production). These comprehensive analyses would establish whether circRNA manipulation alters both metabolic and immune phenotypes in vivo.
*Mechanistic Validation of circRNA-Enzyme Interactions:* For proposed circRNA-enzyme regulatory relationships, RNA immunoprecipitation (RIP) assays should be performed to confirm direct RNA-protein interactions, dual-luciferase reporter assays to validate miRNA binding sites, and mass spectrometry to identify protein complexes associated with candidate circRNAs. These molecular approaches would establish whether proposed circRNA-enzyme interactions are mechanistically valid in chordoma cells.

### 1.9. Potential Immunometabolic Roles

Given the known functions of circRNAs in the immunometabolism of general cancer, it is conceivable that the circRNAs found in chordoma—or others that have not yet been found—also play a part in the disease’s distinct immunometabolic environment. If a circRNA, for instance, stimulates chordoma cell proliferation, it probably does so by affecting metabolic pathways that supply the energy and building blocks required for quick growth. The metabolic status of immune cells within the tumor may also be impacted directly or indirectly by circRNAs that alter the TME in chordoma, such as those that influence immunological checkpoint expression (PD-1, CTLA-4, for example, are enhanced in chordoma) [4].

CircRNAs may play a role in metabolic adaptations and immune evasion. CircRNAs may further give chordoma cells metabolic flexibility in their hypoxic and nutritionally relaxed TME by controlling important enzymes or transporters involved in glucose, glutamine, or lipid metabolism [42]. Furthermore, CircRNAs will alter the actions of immune cells found in the chordoma milieu, such as T cells or macrophages: Their roles may directly impact immunological signaling pathways that result in T cell fatigue or anergy, or they may have an impact on metabolic alterations (e.g., direct macrophage metabolic changes to an M2-like phenotypic shift). One of the main ways that circRNAs coordinate the immunometabolic reprogramming in chordoma may be through their interaction with signaling pathways that are essential for both metabolism and immunity, such as PI3K/AKT/mTOR or NRF2 (Table 1) [43]. To clarify these particular chemical axes in the context of chordoma, more investigation is required.

It is essential to comprehend these particular circRNA-mediated processes in chordoma’s immunometabolism in order to find new diagnostic biomarkers and create focused treatment plans for this difficult cancer.

### 1.10. Scope and Rationale: Bridging Knowledge Gaps in Chordoma CircRNA Immunometabolism

Although circRNAs are increasingly recognized as regulators of cancer immunometabolism, direct evidence in chordoma is almost absent. Current gaps include: (1) lack of comprehensive circRNA profiling across independent chordoma cohorts; (2) no functional studies linking circRNAs to immune or metabolic regulation in chordoma cells or infiltrating immune populations; (3) absence of validated circRNA biomarkers; and (4) no integration of circRNA expression with emerging immune subtypes or the “immune-cold” phenotype of chordoma.

This review synthesizes mechanistic insights from other malignancies to build a theoretical framework for circRNA-driven immunometabolic regulation in chordoma. Chordoma-specific tumor microenvironment features are used to prioritize mechanisms most likely to be relevant, and circTEAD1—the only functionally characterized chordoma circRNA to date—is evaluated as a proof-of-concept for systematic circRNA assessment. Practical experimental directions are outlined to guide the identification and validation of immunometabolism-associated circRNAs, and potential therapeutic implications are discussed in the context of current treatment limitations. This structured approach clarifies the field’s unmet needs and justifies the application of cross-cancer evidence while establishing chordoma-specific research priorities.

### 1.11. Therapeutic Strategies Targeting CircRNAs in Chordoma

Given the emerging role of circRNAs in chordoma, several therapeutic strategies can be envisioned. These strategies can be broadly categorized into direct targeting of circRNAs and indirect targeting through the pathways they regulate [69].

### 1.12. Direct Targeting of CircRNAs

Directly targeting oncogenic circRNAs represents a promising therapeutic avenue. This can be achieved through various approaches, such as antisense oligonucleotides (ASOs) or small interfering RNAs (siRNAs) designed to bind to the back-splice junction of the target circRNA, leading to its degradation [15,16,26]. For example, an ASO targeting circTEAD1 could be developed to reduce its expression in chordoma cells, thereby inhibiting its oncogenic functions. Proof-of-concept for direct circRNA targeting in chordoma has been established through recent experimental studies. In a comprehensive investigation of the circTLK1/miR-16-5p/Smad3 positive feedback axis, Lou and colleagues demonstrated that lentiviral short hairpin RNA (sh-circTLK1) vectors could effectively inhibit circTLK1 expression in human chordoma cell lines U-CH1 and MUG-Chor1 [68]. The study employed multiple functional assays to evaluate the consequences of circTLK1 knockdown: cell proliferation was assessed using both CCK-8 and 5-ethynyl-2′-deoxyuridine (EdU) assays, while cell migration and invasion were quantified through wound-healing assays and Transwell migration/invasion assays. Mechanistically, the authors confirmed that circTLK1 acts as a miRNA sponge for miR-16-5p through dual-luciferase reporter assays, RNA pull-down assays, and RNA immunoprecipitation (RIP) assays, with mass spectrometry identifying Argonaute 2 (AGO2) as the key mediator protein. Functionally, circTLK1 knockdown suppressed chordoma cell proliferation, invasion, and migration, and reversed the epithelial–mesenchymal transition (EMT) phenotype by increasing E-cadherin expression while decreasing N-cadherin and vimentin expression. Notably, the study demonstrated that disruption of the circTLK1/miR-16-5p/Smad3 positive feedback loop via Smad3 inhibitor (SIS3) represents a validated therapeutic approach in chordoma. In vivo validation using a BALB/c nude mouse xenograft model confirmed that U-CH1 cells with circTLK1 knockdown produced significantly smaller and lighter tumors compared to control tumors, with reduced Smad3 expression and decreased EMT progression confirmed by immunohistochemistry (IHC) and Western blotting. These findings establish proof-of-concept that circRNA-targeting strategies can effectively suppress chordoma malignancy through multiple mechanistic pathways.

### 1.13. Indirect Targeting of CircRNA-Regulated Pathways

Indirectly targeting the pathways regulated by circRNAs offers another therapeutic strategy. For example, since circTEAD1 promotes chordoma tumorigenesis by stabilizing Yap1 mRNA, inhibitors of the Yap1/Hippo pathway could be used to counteract the effects of circTEAD1. Similarly, if a circRNA is found to promote glycolysis, inhibitors of glycolytic enzymes could be used in combination with circRNA-targeted therapies (Table 2) [70].

### 1.14. Molecular Crosstalk and Combination Therapies

Because circRNAs, metabolism, and immunology have intricate molecular relationships, combination therapy may be particularly effective. For example, circRNA-targeted therapies may be used in combination with immune checkpoint inhibition or metabolic inhibitors to stop tumor development and overcome resistance mechanisms [79]. Further research is needed to identify the key circRNA-mediated molecular axis in chordoma immunometabolism and to develop effective delivery systems for circRNA-based therapies.

The discovery that circRNAs in chordoma function as both immunological and metabolic network regulators opens up new possibilities for the development of targeted and innovative treatment strategies, which will eventually enhance patient outcomes for this challenging disease.

## 2. Comparative Analysis of Chordoma Molecular and Immunological Studies

A comparison of recent important research that has improved our knowledge of the molecular and immunological landscape of chordoma is given in this section. Contextualizing the function of circRNAs in chordoma immunometabolism requires an understanding of the results presented in these studies (Table 3).

### 2.1. Synthesis and Comparative Insights

The collective findings from these studies paint a picture of chordoma as a molecularly heterogeneous and immunologically complex cancer. The 2015 review by Sun et al. laid the groundwork by establishing the importance of molecular drivers like brachyury and the involvement of non-coding RNAs, specifically microRNAs [7]. This sets the stage for investigating other non-coding RNAs like circRNAs.

The more recent papers build upon this foundation by revealing deeper layers of complexity. The work by Bai et al. (2023) and Zhang et al. (2024) independently converges on the idea of distinct molecular subtypes of chordoma [8,35]. While Bai et al. [8] used gene expression to define subtypes based on chromatin remodeling and developmental pathways, Zhang et al. [35] used proteogenomics to identify subtypes characterized by chromosomal instability and an ‘immune cold’ phenotype. This convergence from different ‘omics’ approaches strongly suggests that these subtypes are biologically meaningful and clinically relevant.

The most critical paper for this review is the 2024 study by Li et al., which is the first to directly implicate a specific circRNA, circTEAD1, in chordoma tumorigenesis [18]. This study is a cornerstone because it not only provides the first direct evidence of circRNA involvement but also links it to a key cancer-related pathway (Hippo/Yap1) and a crucial regulatory mechanism (m6A modification). This provides a mechanistic framework for understanding how circRNAs could contribute to the molecular subtypes identified by Bai et al. [8] and Zhang et al. [35].

Finally, the review by Chen and Zhang (2024) on the immune microenvironment provides the immunological context [5]. It highlights the immunosuppressive nature of the chordoma TME, which is dominated by M2 macrophages and regulatory T cells. This is consistent with the ‘immune cold’ subtype identified by Zhang et al. [35] and provides a potential link between the molecular mechanisms driven by circRNAs and the resulting immune landscape. For example, circRNAs could be involved in the recruitment or polarization of these immunosuppressive immune cells.

Integrating circRNA profiling with immune subtypes in chordoma can be approached through a focused analytical pipeline: subtype-stratified circRNA expression analysis (immune cold vs. immune hot), correlation with established immune markers (CD163/CD206 for M2 macrophages [81]; PDCD1, TIM-3, LAG-3 for T-cell exhaustion [82]), and pathway enrichment of subtype-associated circRNAs based on predicted miRNA interactions [83]. These analyses can be implemented using publicly available bulk RNA-seq datasets (e.g., GEO/EGA) and validated by qPCR in independent cohorts. Where spatial transcriptomic data are available, circRNA patterns could be mapped to regions enriched for immunosuppressive cell states to refine topographic associations. For mechanistic resolution, single-cell circRNA profiling methods [84] would allow linking circRNA expression to macrophage polarization or T-cell exhaustion states at single-cell resolution and enable pseudotime modeling [85]. Collectively, these strategies would help move the field from descriptive associations toward quantitative models that clarify whether specific circRNAs contribute to the immunosuppressive microenvironment characteristic of immune-cold chordoma.

In conclusion, the progression from general molecular characterization to specific circRNA functions and detailed immune profiling demonstrates a growing understanding of chordoma. The next frontier is to integrate these different facets—to understand how specific circRNAs like circTEAD1 contribute to the different molecular subtypes and how they shape the immune microenvironment to promote tumor growth and immune evasion. This review paper is positioned to do exactly that, by synthesizing these findings and proposing a model for circRNA-mediated immunometabolic reprogramming in chordoma.

### 2.2. Prospects of CircRNA-Based Vaccines in Chordoma

CircRNA-based vaccines represent a technically attractive but currently exploratory option for chordoma. Their superior stability, sustained antigen production, and adjustable immunogenicity compared to linear mRNA platforms could, in principle, generate more durable T cell–mediated responses against chordoma-specific targets, thereby addressing the disease’s inherently low immunogenicity and classification as an “immune cold” tumor. Brachyury is a compelling antigen candidate for this approach, given its consistent overexpression in chordoma, restricted expression in normal adult tissues, and prior identification as a target for T cell recognition, making it suitable for circRNA vaccine development in this setting. However, translation remains constrained by several unresolved barriers, including the scarcity of validated tumor antigens beyond brachyury, variability in brachyury expression across tumor subtypes, and practical challenges related to delivery, manufacturing, and safety benchmarking [73]. Consequently, circRNA vaccines should be regarded as a research priority rather than a near-term clinical strategy, contingent upon advances in antigen discovery, immunoengineering, and vector optimization. AI-supported design may accelerate this progress by optimizing circRNA structure, codon usage, and epitope architecture to enhance immunogenicity and therapeutic efficiency [75].

A structured preclinical evaluation framework is essential to advance this concept toward clinical relevance. This process should begin with the construction of circRNA vectors encoding brachyury-derived epitopes optimized for efficient MHC presentation. These constructs would then undergo in vitro validation to confirm antigen presentation and assess T-cell activation in co-culture systems, providing an initial indication of immunogenic potential. Promising candidates should subsequently progress to iterative in vivo testing in xenograft or patient-derived chordoma models to evaluate both immunogenicity and anti-tumor efficacy under physiologically relevant conditions. Collectively, such staged experimentation would provide the foundational evidence necessary to determine whether circRNA vaccines merit advancement to early-phase clinical investigation in chordoma.

### 2.3. Challenges and Future Perspectives

CircRNA-targeted therapies hold significant promise for chordoma, but several critical challenges must be resolved before clinical translation. The most immediate barrier is delivery, particularly given the anatomical complexity of chordoma sites. Skull base tumors require agents that can traverse the blood–brain barrier and avoid neurotoxicity in adjacent cranial nerves and vascular structures, whereas sacrococcygeal tumors pose risks of off-target exposure to the sacral plexus and pelvic organs. These constraints necessitate delivery systems engineered for anatomical selectivity and in vivo testing in orthotopic chordoma models that recapitulate skull base or sacral anatomy. Efficacy studies in these models should be performed in parallel with biodistribution and toxicity assessments using fluorescently or radioactively labeled constructs to clarify the relationship between intratumoral exposure, systemic distribution, and safety.

A second challenge is the risk of unintended molecular perturbation. Because circRNA targeting can influence multiple RNA and protein networks, off-target transcriptome and proteome profiling is essential. Integrating RNA-seq to detect unintended mRNA cleavage or pathway disruption, together with mass spectrometry-based proteomics to assess downstream protein-level effects, will define the therapeutic specificity and reveal potential safety liabilities early in development.

Future progress will depend on four priorities:

1. Engineering delivery vehicles tailored to chordoma anatomy, capable of achieving therapeutic concentrations at tumor sites while minimizing systemic exposure;
2. Establishing standardized pipelines for circRNA detection, quantification, and functional validation across independent chordoma cohorts to ensure reproducibility;
3. Validating immunometabolic circRNA functions in patient-derived organoids and xenografts to determine whether effects on T-cell exhaustion or macrophage polarization are causal;
4. Applying machine learning-based biomarker discovery to predict therapeutic response and identify patient subgroups most likely to benefit.

Overall, translation of circRNA-targeted therapies to the clinic will require anatomically informed delivery innovation, rigorous off-target and toxicity screening, and coordinated multi-institutional efforts. By resolving these challenges, circRNA therapeutics could feasibly emerge as a viable treatment modality for chordoma, a disease for which current systemic options remain limited.

### 2.4. Proposed Preclinical Therapeutic Strategies for circRNA Targeting in Chordoma

In addition to the challenges outlined above, translating circRNA biology into therapy will require concrete preclinical strategies that align with the current molecular landscape of chordoma. I therefore propose four combination approaches designed to generate directly testable hypotheses:

1. circTEAD1 inhibition + Hippo/YAP1 pathway blockade: Using sh-circTEAD1 constructs in combination with verteporfin or XMU-MP-1 in U-CH1/U-CH2 models, with assessment of proliferation, migration, and invasion, followed by xenograft studies to evaluate tumor growth dynamics.
2. circTEAD1 inhibition + metabolic intervention: Combining circTEAD1 suppression with glycolytic modifiers (e.g., 2-deoxy-D-glucose or dichloroacetate), paired with metabolic flux analysis (Seahorse profiling) and subsequent in vivo validation to determine therapeutic synergy.
3. circTLK1 inhibition + TGF-β/Smad axis disruption: Targeting the circTLK1/miR-16-5p/Smad3 axis through SIS3, with EMT marker evaluation (E-cadherin, N-cadherin, vimentin) and functional migration/invasion assays, followed by prioritization of candidates for xenograft validation.
4. circRNA inhibition + immune checkpoint blockade: Preclinical feasibility studies combining circTEAD1 or circTLK1 inhibition with anti-PD-1 or anti-CTLA-4 agents in immune-competent models, expanding to in vivo systems where pharmacologic compatibility can be confirmed through immune profiling and survival endpoints.

Collectively, these strategies provide a disease-focused framework for prioritizing preclinical testing and may help bridge the gap between conceptual therapeutic potential and translational relevance.

## 3. Conclusions

CircRNAs represent a promising new frontier in chordoma biology. The identification of circTEAD1 as an oncogenic driver provides compelling evidence that circRNAs are druggable targets in this rare cancer. However, most proposed immunometabolic roles of circRNAs in chordoma are extrapolated from other cancers and require direct experimental validation.

Several key findings require independent reproduction. The circTEAD1-Yap1 axis has been demonstrated in only two chordoma cell lines and twelve patient samples, and the functional role of circTEAD1 in chordoma-associated immune cells remains unknown. Similarly, the proposed roles of circRNAs in regulating glycolytic enzymes (HK2, PFK), promoting M2 macrophage polarization, and driving T cell exhaustion are based on evidence from other cancers but have not been validated in chordoma.

To move the field forward, a prioritized research roadmap is essential. Comprehensive circRNA profiling across independent chordoma cohorts should be the first priority, followed by functional screening to identify circRNAs with strong effects on chordoma cell behavior and metabolism. Detailed mechanistic studies should then validate the targets and pathways of candidate circRNAs. In vivo validation in relevant preclinical models is critical before considering clinical translation. Finally, chordoma-optimized therapeutic approaches must be developed to address the unique anatomical challenges of skull base and sacrococcygeal tumors.

The therapeutic potential of circRNA-targeted therapies in chordoma is genuine, but realizing this potential requires sustained research efforts that address current knowledge gaps, overcome anatomical and metabolic barriers, and rigorously test safety and efficacy. By maintaining scientific rigor while pursuing innovative approaches, the field can work toward translating circRNA biology into clinical benefit for chordoma patients.

## Figures and Tables

**Table 1:** Immunometabolic Pathways and Their Impact in Various Types of Cancers.

Pathway	Role in Chordoma	Concrete CircRNA	Cancer Type(s)
PI3K/AKT/mTOR	Promotes cell growth, proliferation, and survival; involved in metabolic reprogramming	circPTEN	Breast cancer [44], colorectal cancer [45]
		circAKT1	Cervical cancer [46], renal cell cancer [47]
		circMTOR (regulates mTOR expression)	Hepatocellular carcinoma [48]
NRF2	Regulates antioxidant response and metabolic adaptation; protects against oxidative stress	circKEAP1	Lung cancer [49], osteosarcoma [50]
		circNRF2	Hepatocellular carcinoma [51]
Glycolysis (Warburg Effect)	Provides rapid ATP and metabolic intermediates for biosynthesis; supports tumor growth in hypoxic microenvironment	circHIPK3	esophageal squamous carcinoma [52], breast cancer [53]
		circPVT1	Breast cancer [54], osteosarcoma [55]
		circLDHA	Hepatocellular carcinoma [56]
		circGLIS3	Gastric cancer [57]
Glutaminolysis	Supports amino acid metabolism and biosynthesis; provides nitrogen for nucleotide synthesis	circASS1	Colorectal cancer [58]
Immune Checkpoint Signaling	Contributes to immune evasion by suppressing anti-tumor immune responses; elevated PD-L1 (40–80% of chordomas), CTLA-4, LAG-3, TIM-3	circPVT1	Osteosarcoma [59,60], thyroid cancer [61]
		circPD-L1	Gastric cancer [62], lung cancer [63]
		circLRP6	Prostate cancer [64]
		circIL4R	Hepatocellular carcinoma [65], colorectal cancer [66]
		circCTLA-4	Pancreas cancer [67]
Chordoma-Validated circRNAs		circTEAD1 (circTEAD1/IGF2BP3/Yap1 axis; m6A-dependent mRNA stabilization)	U-CH1, U-CH2 cell lines; xenograft models [34]
		circTLK1 (circTLK1/miR-16-5p/Smad3 positive feedback axis)	U-CH1, MUG-Chor1 cell lines; xenograft models [68]

**Table 2:** Therapeutic Strategies Targeting CircRNAs in Cancers.

Strategy	Mechanism	Examples/Approach	Potential Benefits	Kaynak
Inhibition of oncogenic circRNAs	Degradation or blocking of circRNA function	Antisense oligonucleotides (ASOs), siRNAs, CRISPR-based technologies	Suppress tumor growth, reduce proliferation, overcome drug resistance	[16,69,70,71,72]
Restoration of tumor-suppressive circRNAs	Re-expression or upregulation of circRNA	Gene therapy, small molecules	Inhibit tumor growth, promote apoptosis, enhance chemosensitivity	[73,74]
Indirect targeting via immunometabolism	Modulating circRNAs that influence metabolic pathways or immune checkpoints	Targeting circRNAs regulating glycolytic enzymes, macrophage polarization, immune checkpoint molecules	Reverse metabolic reprogramming, enhance anti-tumor immunity, improve immunotherapy efficacy	[24,75,76,77]
Combination therapies	Combining circRNA-targeted therapies with other treatments	CircRNA inhibition + metabolic inhibitors, CircRNA inhibition + immune checkpoint blockade	Synergistic anti-tumor effects, overcome resistance mechanisms	[78,79]

**Table 3:** Summary of Key Research Findings.

Paper Title	Year	Key Focus	Main Findings	Relevance to circRNA Review
Chordoma: an update on pathophysiology and molecular mechanisms [7]	2015	Pathophysiology, molecular mechanisms	- Brachyury as a key driver - Role of chromosomal alterations, DNA methylation, and microRNAs	Foundational understanding of chordoma molecular biology, including non-coding RNAs.
Gene Expression Profiling Identifies Two Chordoma Subtypes Associated with Distinct Molecular Mechanisms and Clinical Outcomes [8]	2023	Molecular subtyping, clinical outcomes	- Identified two subtypes with distinct molecular mechanisms (chromatin remodeling vs. EMT/Hedgehog pathways) - Subtypes correlate with clinical outcomes	Highlights molecular heterogeneity and the importance of RNA-based classification.
N6-methyladenosine-modified circTEAD1 stabilizes Yap1 mRNA to promote chordoma tumorigenesis [68]	2024	circRNA function in chordoma	- First study on circRNAs in chordoma - circTEAD1 is upregulated and promotes tumorigenesis - m6A modification is crucial for circTEAD1 function - circTEAD1 stabilizes Yap1 mRNA via an RNA-protein complex	Core paper for the review, providing direct evidence of circRNA involvement in chordoma.
Immune microenvironment and immunotherapy for chordoma [5]	2024	Immune microenvironment, immunotherapy	- Chordoma TME is characterized by high infiltration of M2 macrophages and regulatory T cells - Reviews current immunotherapy trials and their limitations	Provides the immunological context for the review, highlighting the immunosuppressive microenvironment that circRNAs may influence.
Proteogenomic characterization of skull-base chordoma [80]	2024	Proteogenomics, chromosome instability	- Chromosome instability is a key prognostic factor - Identified immune cold subtype linked to chromosome 9p/10q loss - Proteomics-based classification reveals subtypes with high CIN and immune cold features	Reinforces the concept of molecular subtypes and links genomic instability to the immune landscape.

## Data Availability

No new data were created or analyzed in this study. Data sharing is not applicable to this article.
